# Chemical shift assignments of calmodulin bound to the GluN1 C0 domain (residues 841–865) of the NMDA receptor

**DOI:** 10.1007/s12104-023-10121-x

**Published:** 2023-02-05

**Authors:** Aritra Bej, James B. Ames

**Affiliations:** Department of Chemistry, University of California, Davis, CA 95616 USA

**Keywords:** CaM, Calcium, GluN1, NMDAR, C0 domain, NMR

## Abstract

Neuroplasticity and synaptic transmission in the brain are regulated by N-methyl-D-aspartate receptors (NMDARs) that consist of hetero-tetrameric combinations of the glycine-binding GluN1 and glutamate-binding GluN2 subunits. Calmodulin (CaM) binds to the cytosolic C0 domain of GluN1 (residues 841–865) that may play a role in the Ca^2+^-dependent inactivation (CDI) of NMDAR channel activity. Dysregulation of NMDARs are linked to various neurological disorders, including Alzheimer’s disease, depression, stroke, epilepsy, and schizophrenia. Here, we report complete NMR chemical shift assignments of Ca^2+^-saturated CaM bound to the GluN1 C0 domain of the human NMDAR (BMRB no. 51715).

## Biological context

N-methyl-D-aspartate receptors (NMDARs) in the brain are localized at the post-synaptic membrane where they regulate neuronal excitability and confer synaptic plasticity (Traynelis et al. 2010). NMDARs contain two copies each of GluN1 and GluN2 subunits, which activate upon binding to the co-agonist glycine and neurotransmitter agonist glutamate, respectively (Benveniste and Mayer 1991; Clements and Westbrook 1991). Under resting basal conditions, the intracellular Ca^2+^ concentration is kept below 100 nM due to the powerful action of Ca^2+^ pumps and exchangers (Berridge et al. 2003; Clapham 2007), and Ca^2+^ sequestration into stores (Berridge 2002; Clapham 2007). Ligand-gated opening of NMDAR channels at the postsynaptic membrane causes intracellular Ca^2+^ levels to increase into the micromolar range (Wadel et al. 2007), causing a wide range of Ca^2+^-dependent processes (Luscher and Malenka 2012; Kunz et al. 2013; Puri 2020). Prolonged elevation of intracellular Ca^2+^ levels is cytotoxic (Stanika et al. 2012), and NMDAR channels are negatively regulated by a process known as Ca^2+^-dependent inactivation (CDI) (Iacobucci and Popescu 2017, 2019, 2020). The Ca^2+^-dependent inactivation of NMDA receptors requires CaM binding to the cytosolic C0 domain in GluN1 (Zhang and Majerus 1998; Iacobucci and Popescu 2017, 2019, 2020). Ca^2+^-free CaM (apoCaM) is believed to be pre-associated with the C0 domain that may cause channel activation at low Ca^2+^ levels (Wang et al. 2008; Iacobucci and Popescu 2017, 2019, 2020). Neurotransmitter binding to NMDAR causes channel opening, which triggers a rise in intracellular Ca^2+^ that promotes a conformational change in the NMDAR/CaM complex, leading to CDI (Krupp et al. 1999; Wang et al. 2008; Iacobucci and Popescu 2020).

Atomic-level structures of NMDARs have been solved by x-ray crystallography (Karakas and Furukawa 2014; Lee et al. 2014) and cryo-EM (Jalali-Yazdi et al. 2018; Regan et al. 2018; Chou et al. 2020) that show detailed inter-subunit interactions between the extracellular ligand-binding domain and transmembrane channel domain. However, the C-terminal cytosolic domain (that mediates CDI) is not visible in any of the published structures and its structure has remained elusive. The cytosolic region of GluN1 (residues 830–938) is comprised of helical domains called C0 (residues 841–865) and C1 (residues 875–898) that interact with CaM (Ehlers et al. 1996; Krupp et al. 1999; Ataman et al. 2007). A crystal structure of CaM bound to the C1 domain has been reported (Ataman et al. 2007), but a structure of CaM bound to C0 is currently not known. We report here NMR resonance assignments of Ca^2+^-saturated CaM bound to the C0 domain of GluN1 (hereafter called CaM/GluN1 C0). These assignments are an important step toward elucidating the complete structure of CaM bound to GluN1, which may be important for understanding the mechanism of CDI.

## Methods and experiments

### Expression and purification of CaM

Human CaM was overexpressed in *E. coli* strain BL21(DE3) using pET11b (Novagen) and the expressed protein was purified as described previously (Bej and Ames 2022a). The CaM protein samples typically have greater than 99% purity as confirmed by sodium dodecyl sulfate–polyacrylamide gel electrophoresis (SDS-PAGE). A peptide fragment of the GluN1 C0 domain of the NMDA receptor (residues 841–865) was purchased from GenScript, dissolved in DMSO-d_6_, and quantified using UV–Vis absorption spectroscopy (ε_280_ = 5500 M^−1^ cm^−1^). A 2.5-fold excess of the peptide was added to Ca^2+^-bound CaM, incubated at room temperature for 30 min, and concentrated to 0.4 mM in a final volume of 0.3 ml.

### NMR spectroscopy

**Fig. 1:**
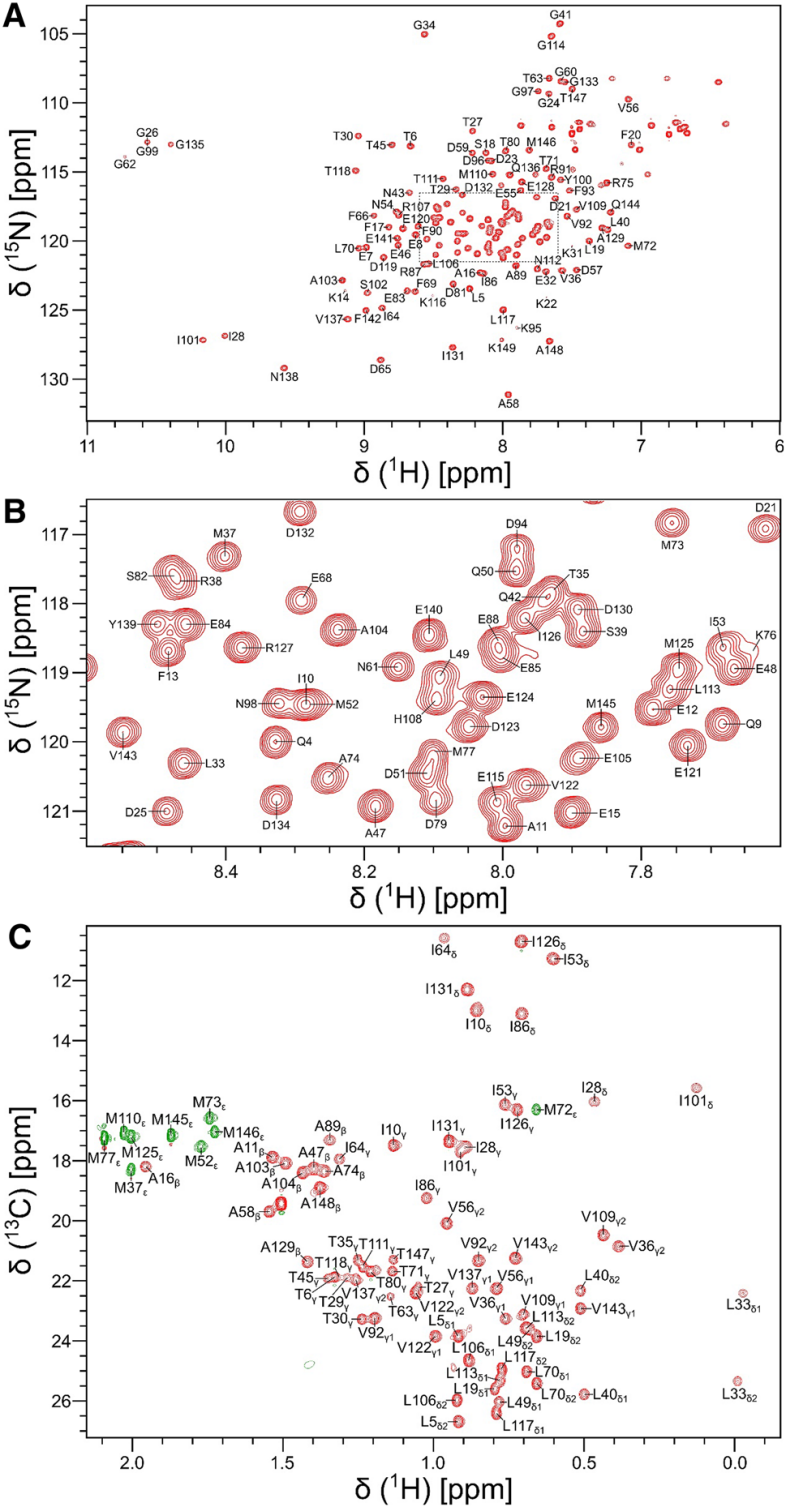
NMR spectra of Ca^2+^-saturated CaM bound to unlabeled GluN1 C0 peptide. **A** ^15^N-^1^H HSQC spectrum recorded at 800 MHz ^1^H frequency. The black-dashed box highlights spectrally crowded region. **B** Expanded view of the resonance assignment from the spectrally crowded region highlighted with black-dashed box. **C** Constant-time ^13^C-^1^H HSQC spectrum of Ca^2+^-saturated CaM bound to the peptide

NMR samples of isotopically labeled CaM bound to the unlabeled GluN1 C0 peptide (complex is called CaM/GluN1 C0) were dissolved in 20 mM Tris-d_11_ (pH 7.0) and 1 mM CaCl_2_ containing 8% (v/v) D_2_O and placed in a Shigemi NMR tube (Shigemi Inc.). All NMR experiments on CaM/GluN1 C0 were performed at 308 K using a Bruker Avance III 800 MHz spectrometer equipped with a triple resonance cryogenic (TCI) probe. The ^15^N-^1^H HSQC spectrum of CaM/GluN1 C0 (Fig. 1A, B) was recorded with 182 × 2048 complex points for ^15^N(F1) and ^1^H(F2). Backbone resonances were assigned by analyzing triple resonance spectra: HNCACB, HN(CO)CACB, HNCO, HBHA(CO)NH, and HBHANH. Side chain resonances (aliphatic (Fig. 1C) and aromatic) were assigned by analyzing HCCCONH-TOCSY, HBCBCGCDHD and HBCBCGCDCEHE as described by (Ikura et al. 1991). The NMR data were processed by NMRPipe (Delaglio et al. 1995) and assignments were obtained using Sparky (Lee et al. 2015).

## Extent of assignments and data deposition

Backbone resonance assignments of CaM/GluN1 C0 are illustrated in the ^15^N-^1^H HSQC spectrum of ^15^N-labeled CaM bound to unlabeled C0 peptide (Fig. 1A, B). Side chain aliphatic resonance assignments are shown by labeled peaks in the constant-time ^13^C-^1^H HSQC spectrum (Fig. 1C). NMR assignments were derived from the analysis of 3D heteronuclear NMR experiments performed on ^13^C/^15^N-labeled CaM bound to unlabeled C0 peptide. The highly dispersed spectral peaks and uniform peak intensities suggest that CaM/GluN1 C0 complex is stable in solution and properly folded. The downfield amide peaks assigned to G26, G62, G99 and G135 indicate that Ca^2+^ is bound to each of the four EF-hands (Fig. 1A). Upfield-shifted side chain methyl peaks assigned to I28, L33, V36, L40, M72, I101, V109 and V143 (Fig. 1C) suggest these residues may interact with aromatic side chain atoms located in the hydrophobic core. At least 97% of the non-proline backbone resonances (^1^HN, ^15^N, ^13^Cα, ^13^Cβ, and ^13^CO) and 97% of side-chain resonances were assigned. Only K78 in the second EF-hand of CaM remains unassigned, because its HSQC peak could not be detected. The chemical shift assignments (^1^H, ^15^N, ^13^C) for CaM/GluN1 C0 have been deposited in the BioMagResBank (http://www.bmrb.wisc.edu) under accession number 51715.

**Fig. 2:**
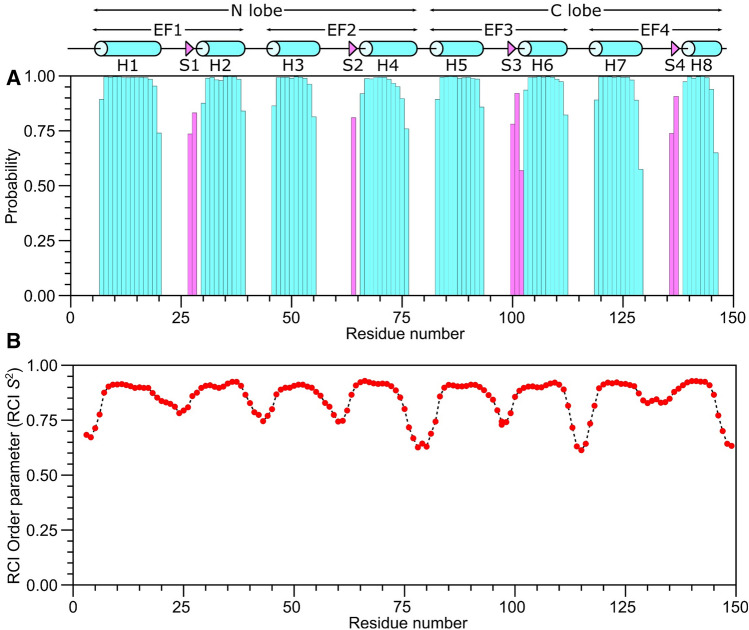
Secondary structure and RCI order parameters (*S*^2^) of Ca^2+^-saturated CaM bound to GluN1 C0 peptide predicted from the assigned backbone chemical shifts. **A** Probability of secondary structural elements (cyan for helix and magenta for strand) and **B** RCI *S*^2^ of Ca^2+^-saturated CaM bound to the GluN1 C0 peptide were predicted using TALOS + server (Shen et al. 2009). The wire diagram depicting the secondary structural elements (cyan cylinder for helix and magenta triangle for strand) was obtained from the CaM structure (PDB ID: 2VAY (Halling et al. 2009))

**Fig. 3:**
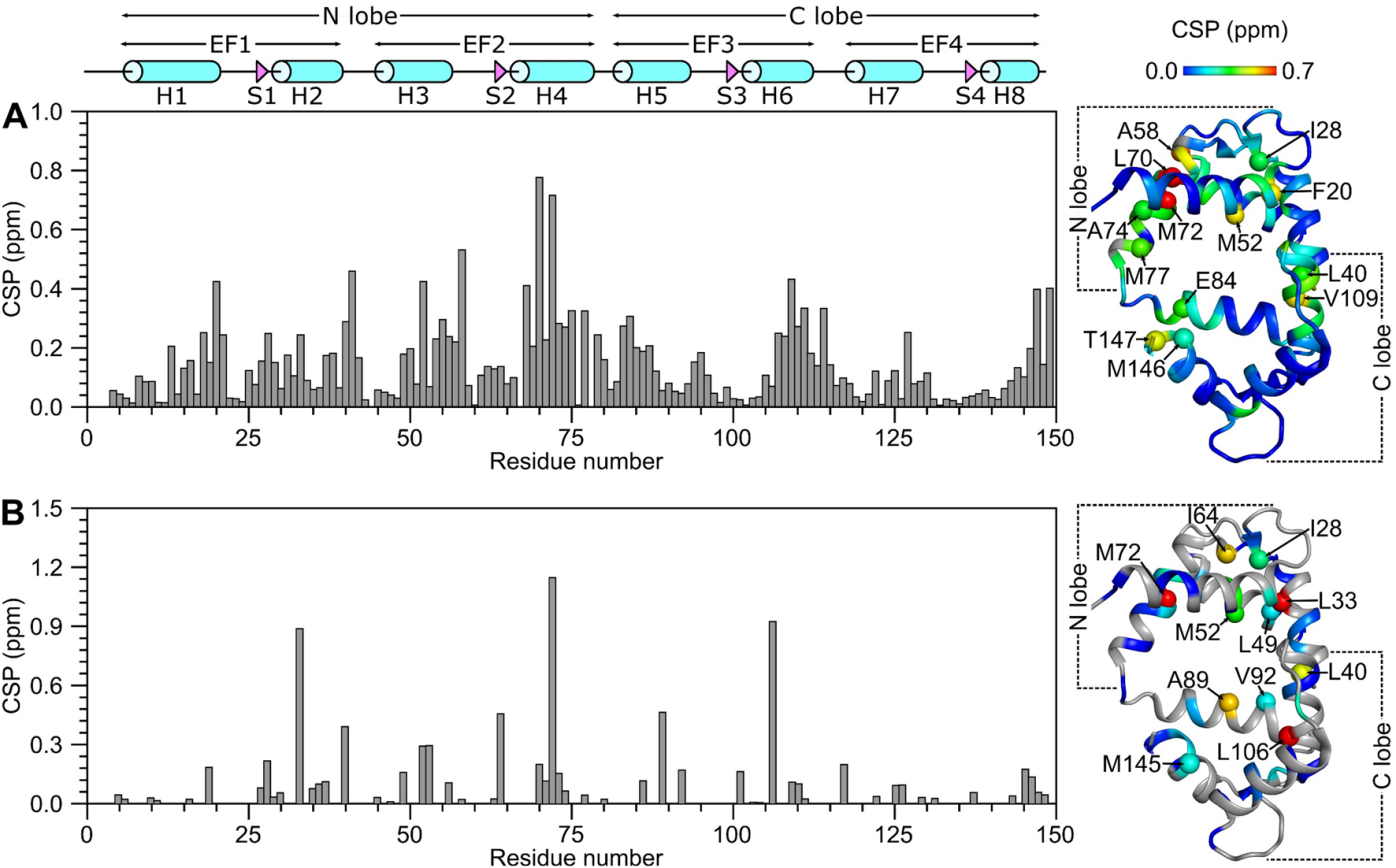
Residue-specific chemical shift perturbation (CSP) for Ca^2+^-saturated CaM in GluN1 C0 peptide-bound and unbound conditions. **A** Backbone amide CSP was calculated as: $CSP= \sqrt{{\left(\Delta {H}^{N}\right)}^{2}+{\left(0.14\times \Delta N\right)}^{2}}$. ΔH^N^ and ΔN are the observed difference in the ^1^H^N^ and ^15^N chemical shifts, respectively between peptide-bound and unbound Ca^2+^-saturated CaM (Bej and Ames 2022b). **B** Side-chain methyl CSP was calculated as: $CSP= \sqrt{{\left(\Delta H\right)}^{2}+{\left(0.14\times \Delta C\right)}^{2}}$. ΔH and ΔC are the observed difference in the ^1^H and ^13^C methyl chemical shifts, respectively between peptide-bound and unbound Ca^2+^-saturated CaM. CSP values are mapped on to the CaM structure (PDB ID: 2VAY (Halling et al. 2009)). Residues with significant CSP are showed in spheres and labeled accordingly. Residues, without CSP values including proline, amino acids without methyl group, or unassigned resonances, are colored as gray

The CaM/GluN1 C0 secondary structure was calculated based on chemical shift index (Wishart et al. 1992) and ANN-Secondary structure prediction using TALOS + (Shen et al. 2009) (Fig. 2). The CaM/GluN1 C0 secondary structure is similar to that reported previously for free CaM (Bej and Ames 2022b), and is depicted by cylinders and triangles in Fig. 2A. The analysis of sequential NOE patterns along with long-range NOE-derived distances identify a total of eight α-helices and four β-strands that make up four EF-hands (EF1: residues 7–39, EF2: residues 45–76, EF3: residues 83–112 and EF4: residues 119–144) as seen in the crystal structure of Ca^2+^-bound CaM (Babu et al. 1988). Two N-terminal EF-hands (EF1 and EF2) form the CaM N-lobe, and C-terminal EF-hands (EF3 and EF4) form the C-lobe. The GluN1 C0 peptide binds to CaM and causes chemical shift perturbations (CSPs) for hydrophobic CaM residues in the N-lobe (F20, I28, L33, L40, L49, M52, A58, M72) and C-lobe (A89, V92, L106, V109, M145, M146) (Fig. 3A, B), suggesting that the GluN1 C0 peptide is making contact with both lobes of CaM as seen in previous collapsed structures of CaM bound to other peptides (Hoeflich and Ikura 2002).

**Fig. 4:**
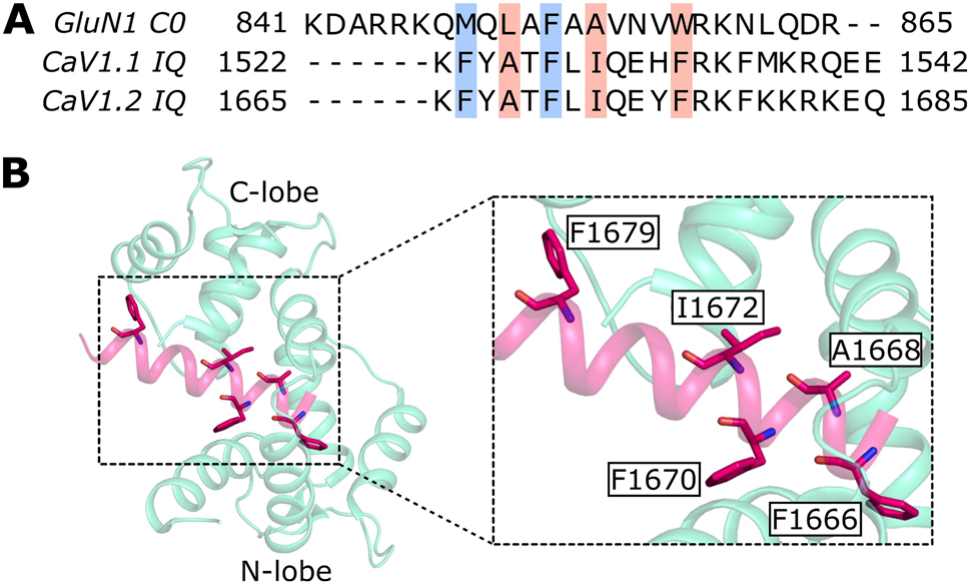
Conserved residues in GluN1 C0 that may contact CaM. **A** Sequence alignment of GluN1 C0 with the IQ motif of CaV1.1 and CaV1.2. Residues contacting the N-lobe and C-lobe of CaM are highlighted in blue and red, respectively. **B** Crystal structure of CaM bound to CaV1.2 IQ peptide (PDB: 2F3Y (Fallon et al. 2005)). Key residues involved in interaction with CaM are labeled

The amino acid sequence of GluN1 C0 when aligned with the IQ-motif of L-type Ca^2+^ channels (CaV1.1 and CaV1.2) reveal critical conserved hydrophobic residues that likely contact CaM (Fig. 4A). The GluN1 C0 residues L850, A854 and W858 align with conserved hydrophobic residues of the IQ-motif that contact the CaM C-lobe (highlighted red in Fig. 4A) as seen in the known structures of CaM bound to the IQ-motif of CaV1.2 (Fig. 4B) (Fallon et al. 2005) and CaV1.1 (Halling et al. 2009). Therefore, we predict that these residues in GluN1 C0 likely contact the CaM C-lobe. In addition, the GluN1 C0 residues M848, A851 and F852 align with conserved hydrophobic residues (highlighted blue in Fig. 4A) that contact the CaM N-lobe in the crystal structure of CaM bound to the CaV1.2 IQ peptide (Fig. 4B), suggesting that these residues from GluN1 C0 may contact the CaM N-lobe. Future NMR studies are needed to determine the NMR structure of CaM/GluN1 C0 and test whether it is similar to the collapsed structure of CaM bound to CaV1.2 IQ (Fig. 4B). The NMR assignments of CaM/GluN1 C0 presented here are an important first step toward determining the full three-dimensional structure of CaM bound to GluN1 C0.

## Data Availability

The assignments have been deposited to the BMRB under the accession code: 51715.
